# Genotoxicity and Cytotoxicity Assessment of Volatile Organic Compounds in Pathology Professionals Through the Buccal Micronuclei Assay

**DOI:** 10.3390/toxics13050411

**Published:** 2025-05-19

**Authors:** Fátima Baptista, Patrícia V. Garcia, Armindo S. Rodrigues, Carina Ladeira

**Affiliations:** 1 FCT—Faculty of Sciences and Technology, University of the Azores, 9501-801 Ponta Delgada, Portugal; 2022103583@uac.pt (F.B.); patricia.v.garcia@uac.pt (P.V.G.); armindo.s.rodrigues@uac.pt (A.S.R.); 2 cE3c—Centre for Ecology, Evolution and Environmental Changes & CHANGE—Global Change and Sustainability Institute, Azorean Biodiversity Group, University of the Azores, 9501-801 Ponta Delgada, Portugal; 3 IVAR, Institute of Volcanology and Risks Assessment, University of the Azores, 9501-801 Ponta Delgada, Portugal; 4 H&TRC—Health & Technology Research Center, ESTeSL—Escola Superior de Tecnologia da Saúde, Polytechnic University of Lisbon, 1990-096 Lisbon, Portugal; 5 NOVA National School of Public Health, Public Health Research Centre, Universidade NOVA de Lisboa, 1600-560 Lisbon, Portugal; 6 Comprehensive Health Research Centre (CHRC)—Pólo ENSP, 1600-560 Lisbon, Portugal

**Keywords:** DNA damage, buccal mucosa cells, micronuclei, volatile organic compounds, pathology lab, occupational exposure

## Abstract

In pathology laboratories, several volatile organic compounds (VOCs) are used, such as formaldehyde, ethanol, and xylene. These substances are recognized as genotoxic and cytotoxic, which is why their handling poses risks to human health. The buccal micronucleus (MN) cytome assay is a non-invasive, useful, and simple method to detect these effects in exposed individuals. The aim of the study was to evaluate the risk of genotoxicity and cytotoxicity of VOCs in pathology professionals of S. Miguel Island, Azores, Portugal. The study comprised two groups: exposed workers (n = 21) from the three laboratories of S. Miguel, and a reference group (n = 50), randomly chosen from other hospital services without known exposure to VOCs. The exfoliated buccal cells were auto-sampled by all the participants using a cytobrush. The samples were processed in ThinPrep^®^, stained with modified Feulgen with Fast Green, and visualized for MN and other nuclear anomalies (ONAs), such as karyorrhexis, pyknotic, and karyolytic cells. Results showed that VOCs have a predictive significance for MN frequency, leading to the conclusion that their exposure is an increased risk factor for the health of these professionals, approximately four times greater than in the control group.

## 1. Introduction

In pathology anatomy laboratories, several chemical substances considered toxic, carcinogenic, mutagenic, and teratogenic, such as formaldehyde and xylene, are widely used routinely. Additionally, the mixture with other chemicals also used in the laboratory creates a very diverse chemical environment, with incalculable risks to human health [1,2]. Formaldehyde is the universal fixative used in histopathology, and it is classified by the International Agency for Research on Cancer (IARC) as carcinogenic to humans [3]. Xylene is one of the most used solvents in pathology laboratories as a clearing agent to prepare tissues for microscopic analysis. The main health effects of exposure to xylene are associated with the central nervous system [4,5]. Long-term exposure to solvent vapors often occurs in mixtures rather than a single solvent, leading to nervous system effects at higher concentrations.

Decree-Law No. 39/2018, of 11 June [6], establishes the European Union’s policy, in accordance with the values of the World Health Organization (WHO) and specifically refers to facilities that use volatile organic compounds (VOCs), suggesting precautions regarding the need not to exceed the permitted exposure values that have been proven to cause irreparable human damage. In this context, they warn of the replacement of some chemicals with others with less aggressive properties in relation to the classification as carcinogenic, mutagenic, or toxic for reproduction (CMR).

Buccal mucosa provides a barrier to xenobiotics that can be metabolized into potential reactive products. As up to 90% of all cancers have an epithelial origin, the buccal mucosa could be used to monitor early genotoxic events [7]. Therefore, oral mucosal epithelial cells are used in micronucleus (MN) cytome assay for early detection of genotoxic events, with the main routes of contact via ingestion and inhalation. Thus, the frequency of MN is a very sensitive endpoint for detecting early changes in DNA [8], including those resulting from occupational exposure to VOCs [9]. Biomonitoring studies in human populations, using biomarkers of genotoxicity such as MN and cytotoxicity such as other nuclear abnormalities (ONAs), namely karyorrhexis, karyolysis, and pyknosis, are useful tools for assessing genetic risks resulting from exposure to chemicals, as they allow the assessment of DNA damage [10,11,12].

The buccal mucosa is a stratified squamous epithelium consisting of four distinct layers, such as *stratum corneum*, or keratinized cell layer, lines the oral cavity comprising cells that are constantly being shed as a result of wear and tear of surface tissue, below the *stratum granulosum*, or granular cell layer, and the *stratum spinosum*. Beneath these layers are the *rete pegs* or *stratum germinativum*, containing actively dividing basal cells and basal stem cells, which produce progeny that differentiate and maintain the profile, structure, and integrity of the buccal mucosa [7,9]. Consequently, the detection of increased MN frequencies in exfoliated buccal cells requires that a genotoxic agent overwhelms the permeability barrier, reaches the basal layer on paracellular and/or transcellular routes, and induces DNA lesions that become MN during cell division. These cells then have to migrate to the surface, within 21 days, to be collected for the MN test [10,13].

According to Viegas et al. [14] and Ladeira et al. [15], occupational exposure to formaldehyde in workers in pathology anatomy laboratories resulted in higher MN values in exposed individuals in comparison with non-exposed, with a specific association with the macroscopic examination task. Similar results were found in the studies of Goyer et al. [16] and Orsière et al. [17] are systematized in the review by Fenech et al. [18].

In regard to xylene, the study from de Aquino et al. [19] in pathology laboratory technicians showed a higher frequency of karyolytic and apoptotic-like cells (karyorrhectic and pyknotic) in relation to the control group; however, regarding buccal cell MN, no statistical significance was found between the groups. On the contrary, the study from Ladeira et al. (2020) showed higher levels of MN and nuclear buds quantified by the CBMN assay, and also higher DNA damage and oxidative DNA damage measured by the comet assay [20].

Human biomonitoring studies of occupational exposure to VOCs, which included formaldehyde and xylene combined, verified a statistically significant increase in MN in buccal cells of the exposed group in comparison with [21,22]. However, the studies from De Oliveira et al. [23] and Ferri et al. [24] found no significant differences between the groups.

Recently, Sommer et al. (2020) reinforced the importance of using MN to assess genotoxicity in human biomonitoring programs, highlighting the importance of developing and using automatized methodologies [25]. Oral exfoliative cytology and liquid cytology techniques have clinical validation for diagnosis and allow the observation of epithelial cell morphology and contribute to the study of cytomorphometry [26,27]. It was found that the MN assay in human buccal cells has not yet been tested in monolayer cytology in the context of evaluating genotoxicity and cytotoxicity simultaneously in workers in pathological anatomy laboratories, despite the high level of exposure to toxic substances in the occupational context. On the other hand, the development of rapid and effective cytological procedures that allow timely assessment of the risks of this exposure is crucial for biomonitoring and the development of preventive measures in this professional activity.

This study aimed to evaluate the genotoxic and cytotoxic effects of occupational exposure to VOCs in pathology anatomy workers, in comparison with a reference group without exposure, by using a non-invasive method such as the exfoliated buccal cells MN cytome assay.

## 2. Materials and Methods

### 2.1. Participants and Samples

A total of 71 individuals participated in the study as follows: 21 workers from the 3 laboratories that exist in S. Miguel (Azores, Portugal) occupationally exposed to VOCs, and 50 individuals without exposure to VOCs recruited from other hospital services. All participants signed an informed consent and completed a questionnaire with the aim of characterizing demographically the participants. The study was approved by the Ethics Committee of the University of Azores (reference No. 59/2023) and the Ethics Committee of Hospital Divino Espírito Santo (reference No. S-HDES/2024/29).

### 2.2. Exfoliated Buccal MN Cytome Assay

Exfoliated buccal epithelial cells were self-sampled, with a cytobrush, from the inside of both cheeks to maximize cell sampling and to eliminate any unknown biases that may be caused by sampling one cheek only. With that purpose, all the participants received 1 brush in sterile conditions, and after making 5 rotations with it in each inner cheek (right and left), the brushes were immersed in Thinprep^®^ fixation solution (PreservCyt^TM^, CAS: 65-56-1, Hologic, Marlborough, MA, USA) and kept in a specific Thinprep^®^ flask to be transported to the laboratory and processed. Each flask was processed in monolayer cytology equipment ThinPrep^®^ 2000 (Hologic, Marlborough, MA, USA) according to the manufacturer’s instructions.

Two glass slides were prepared for each individual and immersed in 96% ethanol before the start of the staining technique. The slides were treated with 5M HCl (CAS: 7947-01-0, Fluka, Honeywell, Seelze, Germany) for 10 min and with Schiff’s reagent (Bio-Optica, Milan, Italy) for 45 min in a dark room. After tap water washing for 3 min, the slides were washed in distilled water and stained for 10 s with Fast Green 0.25% (CAS: 5141-20-8, Sigma-Aldrich, St. Louis, MO, USA), dehydrated (ethanol at 96% and 100%), and coverslipped in CV5030 equipment (Leica, Wetzlar, Germany).

### 2.3. Visualization and Scoring

**Figure 1:**
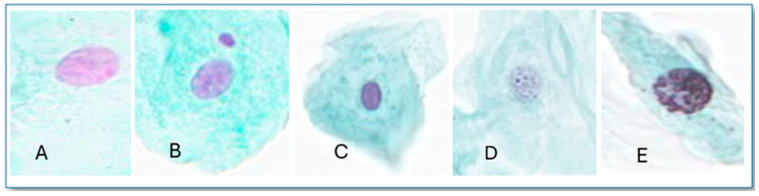
Buccal exfoliated cells stained by the modified Feulgen technique and Fast Green that represent (**A**) normal cell, (**B**) cell with MN, and ONAs, (**C**) pyknosis, (**D**) karyolysis, and (**E**) karyorrhexis. Magnification: 1000× with immersion oil.

The slides were visualized by a single expert, blindly, under a Leica CM Optical Microscope (Leica Biosystems, Nussloch, Germany) at 400× magnification. Two thousand cells were visualized from each individual, and cells with MN and other nuclear abnormalities (ONAs), namely karyorrhexis, karyolysis, and pyknosis (Figure 1), were scored according to Tolbert et al. [28] and Thomas et al.’s [29] criteria for exfoliated buccal mucosa cells.

### 2.4. Statistical Analysis

All data were analyzed using IBM Statistical Package for Social Sciences (SPSS) 29.0.0 software, and the level of statistical significance was set at *p* < 0.05. Descriptive analysis was performed to address demographic sample characteristics and arithmetic mean ± standard deviation (SD). All the outlier values were included in the statistical analysis. The Shapiro–Wilk test was used to assess the normality of the sample distribution. According to that, qualitative data were analyzed by the chi-square (X^2^) test and quantitative data by Mann–Whitney U test. Spearman correlations were used to test the MN association with the demographic variables (age, gender, alcohol consumption (yes vs. no), tobacco consumption (yes vs. no), and use of mouthwash (yes vs. no)). The relative risk (RR) between MN and VOC exposure (estimated by the exponential of the regression coefficient of the corresponding variable), with 95% confidence intervals, was estimated using a generalized linear model (Poisson Regression Model), adjusted for age, gender, the use of mouthwash (yes vs. no), consumption of tobacco (yes vs. no), and consumption of alcohol (yes vs. no).

## 3. Results

The mean age of the exposed workers (n = 21) was 39.38 ± 2.60 years, 76.2% (n = 16) being females and 23.8% (n = 5) being males. For the control group (n = 50), the mean age was 47.72 ± 1.53 years, with 84% (n = 42) females and 16% (n = 8) males. Table 1 shows lifestyle factors such as tobacco and alcohol consumption, and mouthwash use frequency from both groups. Statistically significant differences between groups were not found, with the exception of age (*p* = 0.009, Mann–Whitney test), being the control group older than the exposed group.

### 3.1. Genotoxicity and Citotoxicity Assessment

For the genotoxicity (MN) and cytotoxicity (ONA) biomarkers studied in the buccal cells, an increase was found in all the endpoints in the exposed workers in comparison with controls. Despite this, statistically significant differences were found for MN and karyorrhexis (*p* < 0.05), and not for pyknosis and karyolysis.

#### 3.1.1. Micronucleus (MN)

**Figure 2:**
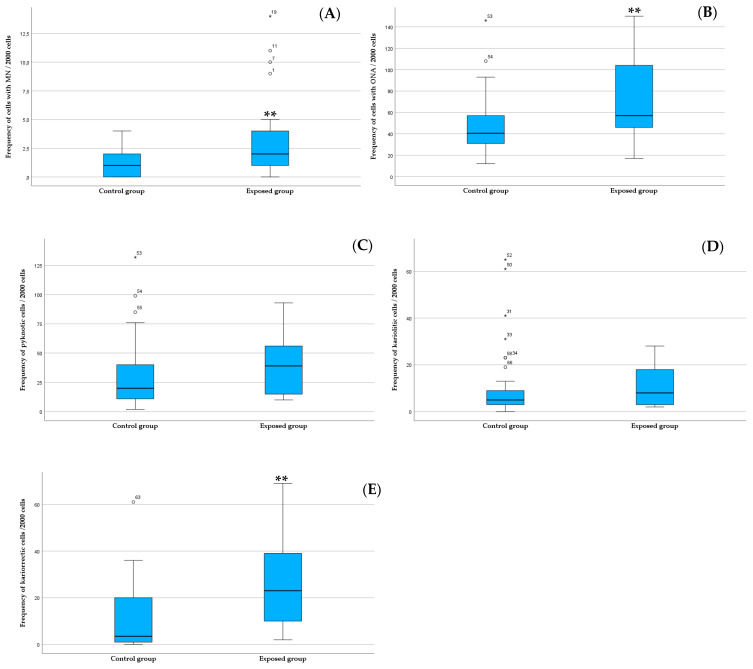
Boxplots of MN (**A**), ONA (**B**), pycknosis (**C**), karyolysis (**D**), and karyorrhexis (**E**) medians, minimum and maximum values, in exposed and controls. ° represents outliers and (*) extreme outliers; ** represents significant differences between the two groups (Mann–Whitney U test, *p* ≤ 0.05).

The results showed statistically significant differences between workers exposed to VOCs (M = 1) in comparison with controls (M = 2) (*p* = 0.002, Mann–Whitney U test; Figure 2A). The mean frequency (±SE) of cells with MN was 3.62 ± 0.86 (exposed group) and 1.14 ± 0.16 (control group).

Age and sex are considered the most important demographic variables affecting MN frequency. Since it was found statistically significant differences regarding age between workers exposed (39.38 ± 2.60) and controls (47.72 ± 1.53), the association between age and MN frequency was determined. No association was found between MN frequency and age or with any of the other variables (sex, tobacco consumption, alcohol consumption, and mouthwash use), either for all the subjects or for each group when analyzed separately (Table 2).

#### 3.1.2. Other Nuclear Abnormalities (ONAs)

In general, ONAs presented higher values in exposed (M = 57.0) in comparison with controls (M = 40.5), being that difference being statistically significant (*p* = 0.005, Mann–Whitney U test; Figure 2B). The mean frequency (± SE) of cells with ONAs was 74.81 ± 8.56 (exposed group) and 48.96 ± 3.92 (control group). Regarding pyknosis, it was found to be higher in exposed (M = 20) in comparison with controls (M = 20), although such a difference was not statistically significant (*p* = 0.05, Mann–Whitney U test; Figure 2C). The mean frequency (± SE) of pyknotic cells was 38.90 ± 5.25 (exposed group) and 28.56 ± 3.79 (control group). Karyolysis was slightly higher in exposed (M = 8) in comparison with controls (M = 5), without statistical significance (*p* = 0.201, Mann–Whitney U test; Figure 2D). The mean (± SE) of karyolytic cells was 10.76 ± 1.79 (exposed group) and 9.60 ± 1.91 (control group). Finally, regarding karyorrhexis, higher mean values were found in exposed (M = 23) in comparison with controls (M = 3.5), that difference being statistically significant (*p* < 0.001, Mann–Whitney U test; Figure 2E). The mean (±SE) of karyorrhectic cells was 25.14 ± 3.94 (exposed group) and 10.80 ± 1.93 (control group).

### 3.2. Genotoxicity Risk Assessment

Occupational exposure to VOCs in pathology anatomy workers was revealed to be a significant predictor of the frequency of micronucleated cells in the multivariate analysis. After adjustment for age, use of mouthwash, tobacco consumption, and alcohol consumption, a higher risk for an increased frequency of micronucleated cells was found associated with the occupational exposure to VOCs (RR = 3.77; 95% CI, 2.52–5.65; *p* < 0.001). Among the analyzed confounding factors, only age showed a significant effect regarding the frequency of micronucleated cells (Table 3).

## 4. Discussion

Most research on the effects of chemicals on biologic systems is conducted on one chemical at a time. However, in the real world, people are exposed to mixtures, not single chemicals [30,31]. VOCs are known to have carcinogenic, mutagenic and reproductive effects [32], capable of causing changes in DNA, such as the formation of MN and ONAs, and it is therefore expected that these effects would be more evident in the group exposed to the environment of the pathology laboratories compared to the reference group.

Regarding demographic characteristics, it was found that the average age of the exposed group was significantly lower (39.38 ± 2.60) than that of the controls (47.72 ± 1.53). However, it was found that there is no significant association between MN and the age of the participants. This fact contradicts some studies that point to age as a promoter of MN [10] and corroborates others where the frequency of MN did not increase significantly with age [9,33,34]. Cytogenetically, aging is associated with a series of serious cellular changes, including size, morphology, and changes in cell expression and proliferation [35,36]. The influence of age on genotoxic and cytotoxic endpoints possibly reflects the increase in spontaneous chromosome instability with aging, associated with an accumulation of DNA damage due to a progressive impairment of overall DNA-repair capacity [37]. The lack of association between MN and age could be explained by the short age range of the participants in our study.

For categorical variables, such as sex, alcohol consumption, tobacco consumption, and use of elixir, no significant differences were obtained between the groups. Similar results for these confounding variables were recently published by Linhares et al. [38], Caponio et al. [39], and Garcia et al. [9] regarding occupational exposure of thermoelectric power-plant workers to VOCs. The lack of differences between the two groups is considered relevant, since the variables can affect the frequency of MN in oral cells, and therefore, should be controlled, as reported by Holland et al. [10], Bonassi et al. [40,41], Bolognesi et al. [42], and Ramos et al. [43]. The study from Nersesyan et al. (2006), which compared different MN stains in buccal samples from heavy tobacco smokers and controls, reported that exposure of oral mucosa cells to genotoxic agents, lead to the induction of micronuclei in these cells [44], and also the study from Çelik et al. [45] found a significant increase in the frequency of MN in the buccal epithelial cells of workers exposed to VOCs who smoked. More recently, de los A. Gutiérrez et al. (2020) found that smoking habit represented a significant factor increasing MN in the inhabitants of urban and industrial areas (the latter area with high levels of air pollutants, particularly VOCs) from Buenos Aires [46].

In relation to VOC exposure and the frequency of cells with MN, it was found that in the exposed group, it was 3.62 ± 0.86, and in the unexposed group, it was 1.14 ± 0.16 per 2000 buccal epithelial cells analyzed. The frequency of MN in the exposed group is above the normal range defined by Bonassi et al. (2011) for human oral epithelia (0.3–1.7 MN/1000 cells) while the values observed for the control group are within the range of these basal values [40]. Comparing these results with the reference values of the spontaneous frequency of cells with MN, where 0.3–1.7 MN/1000, meaning a range of 0.6–3.4 per 2000 cells and standard deviation of 0.32 and 1.70, we can observe that our data fit these values, whether from controls or the exposed group, although there was a significant increase in the frequency of micronucleated cells in the exposed group [40]. Holland et al. (2008) report that the most reported frequencies are between 0.05 and 11.5 per 1000 cells, i.e., (0.10–23), with most values between 0.5 and 2.5 per 1000 cells, i.e., 1–5 in 2000 cells [10]. Other studies present similar results, such as those by Akbar-Khanzadeh et al. [47], Ladeira et al. [15,20,30], Ramos et al. [43], and de Aquino et al. [19]. Despite this, higher MN values were observed in the group of exposed individuals, denoting the preponderance of exposure. An example where the frequency of micronucleated cells was significantly higher in the exposed group (5.26 vs. 1.33 MN/2000 cells), being above the reference values indicated by Bonassi et al. [40], and reported by [8]; nevertheless, in this study, the genotoxic agent was ionizing radiation. Also, Sivasankari et al. (2020) study states that the frequency of MN is higher in the group that had more [48].

The results also revealed a significant increase in the frequency of cells with ONAs, being 74.81 ± 8.56 for the exposed group and 48.96 ± 3.92 for the controls. VOCs can produce effects on the body long after exposure has occurred. This fact may explain the cytotoxic effect found [19]. To analyze these values, the frequency distribution among the ONA cells studied was compared, namely pyknosis, karyolysis, and karyorrhexis. In relation to pyknosis, a mean of 38.90 ± 5.25 was obtained for the exposed group compared to 28.56 ± 3.79 in the controls, although such a difference was not statistically significant. Regarding karyolysis, this value was similar between the groups, i.e., 10.76 ± 1.79 and 9.60 ± 1.91, exposed and controls, respectively, without statistically significant differences. However, karyorrhexis stood out in ONAs, with a significantly higher frequency in the exposed group (25.14 ± 3.94) than in controls (10.80 ± 1.93). It is important to note that those cells in karyorrhexis appear in advanced stages of necrosis and apoptosis [49,50], generally associated with severe DNA damage. In this study, exposure to VOCs was shown to have a significant effect on the frequency of cells with MN in the regression model used. After adjusting for alcohol consumption, tobacco, and use of mouthwash, a four-fold increase in risk of formation of micronucleated cells associated with exposure to VOCs was observed (RR = 3.77; 95% CI, 2.52–5.65; *p* < 0.00), linking with a higher risk of carcinogenesis than the control group. Several studies refer to the reagents used in pathology anatomy laboratories, classifying them as serious risk enhancers for human health over the years. While formaldehyde exposure effects are already well characterized [47,51,52], effects of xylene exposure on human health are still controversial, and increasing attention has been given to occupational exposure [53]. In this sense, the present work assessed possible adverse effects in workers occupationally exposed to formaldehyde, xylene, and other volatile organic compounds, and the DNA damage and cytotoxic effects in these individuals. According to Holland et al. [10], buccal epithelial cells represent a recognized target site for early genotoxic events induced by carcinogenic substances that enter the body via inhalation or ingestion. Torres et al. (2019) report that the frequency of cells with MN in the oral epithelium is a highly sensitive biological dosimeter and can be used to detect early DNA damage caused by exposure even to low doses of ionizing radiation, adding the advantage that is a sample collected by a non-invasive method [8]. In this study, the monolayer cytology technique was applied, ThinPrep^®^ by Hologic, routinely used in in vitro diagnosis methods for diagnosis purposes, which met the quality parameters for good observation of nuclear alterations, being an excellent alternative to smears or other laboratorial procedures.

## 5. Conclusions

In summary, our results showed a significant association between occupational exposure to VOCs and the occurrence of MN and some ONAs (particularly karyorrhexis) in buccal epithelial cells of pathology anatomy laboratory workers occupationally exposed to these substances. Despite existing legislation and combined protection measures, this research showed the need for biomonitoring workers, as well as investment in facilities to mitigate this hazardous work environment. Through the buccal micronucleus test, it is possible to guide and validate high-quality biomonitoring indicators and assess the genotoxic and cytotoxic effects in pathology laboratories, where VOC occupational exposure is a reality. Hopefully, this research can contribute scientific evidence-based knowledge for the risk of exposure of these professionals and serve as a driver for measures.

## Figures and Tables

**Table 1:** Demographic characteristics and lifestyle habits of both groups—exposed and controls.

	Exposed Group (n = 21)	Control Group (n = 50)	*p*-Value ^1^
Years (mean ± SD)	39.38 ± 2.60	47.72 ± 1.53	0.009 *
Sex			
Males	5 (23.8%)	8 (16%)	0.437
Females	16 (76.2%)	42 (84%)	
Tobacco consumption			
Yes	5 (23.8%)	10 (20%)	0.720
No	16 (76.2%)	40 (80%)	
Alcohol consumption			
Yes	15 (71.4%)	27 (54%)	0.173
No	6 (28.6%)	23 (46%)	
Mouthwash use			
Yes	8 (38.1%)	27 (59%)	0.221
No	13 (61.9%)	23 (46%)	

^1^ *p*-value: Mann–Whitney U test for years and X^2^ test for tobacco, alcohol, and mouthwash use. * *p* < 0.05.

**Table 2:** Spearman correlations between MN and age, sex, tobacco and alcohol consumption, and mouthwash use in all subjects, exposed and controls (C—correlation coefficient).

	All (n = 71) (C; *p*-Value)	Exposed (n = 21) (C; *p*-Value)	Controls (n = 50) (C; *p*-Value)
Age	−0.021; 0.861	0.303; 0.181	0.141; 0.328
Sex	−0.079; 0.514	−0.067; 0.774	−0.142; 0.324
Tobacco consumption	−0.28; 0.818	0.10; 0.967	−0.053; 0.717
Alcohol consumption	0.026; 0.830	0.206; 0.369	−0.118; 0.416
Mouthwash use	0.041; 0.734	−0.117; 0.614	0.174; 0.226

**Table 3:** Adjusted association between characteristics of study participants, occupational exposure to VOCs, and the frequency of MN.

Poisson Regression (GLZ)	N: 71 Prob ˃ χ2 ˂ 0.001		
	N (%)	RR (95% CI) ^a^	*p*-Value
Age		1.03 (1.01–1.04)	0.002 *
Gender			
Male	13 (18.3)	0.76 (0.48–1.20)	0.236
Female	58 (81.7)	1	
Mouthwash use			
Yes	35 (49.3)	1.03 (0.71–1.47)	0.896
No	36 (50.7)	1	
Tobacco consumption			
Yes	15 (21.1)	1.24 (0.83–1.83)	0.293
No	56 (78.9)	1	
Alcohol consumption			
Yes	42 (59.2)	1.30 (0.88–1.93)	0.188
No	29 (40.8)	1	
Occupational exposure to VOCs			
Exposed group	21 (29.6)	3.77 (2.52–5.65)	<0.001 *
Control group	50 (70.4)	1	

^a^ RR, relative risk, 95% CI, 95% confidence interval; * significance level set at *p* < 0.05.

## Data Availability

Data can be available upon request.

## Abbreviations

The following abbreviations are used in this manuscript:

IARC	International Agency for Research on Cancer
MN	Micronucleus
ONAs	Other nuclear abnormalities
VOCs	Volatile organic compounds
RR	Relative risk
